# Epidemiological study on factors influencing the occurrence of helminth eggs in horses in Germany based on sent-in diagnostic samples

**DOI:** 10.1007/s00436-022-07765-4

**Published:** 2023-01-11

**Authors:** Heike Boelow, Jürgen Krücken, Georg von Samson-Himmelstjerna

**Affiliations:** Institute for Parasitology and Tropical Veterinary Medicine, Freie Universität Berlin, Robert-Von-Ostertag-Str. 7, 14163 Berlin, Germany

**Keywords:** Strongyles, *Parascaris* spp., Mini-FLOTAC, Combined sedimentation/flotation, Equine parasites, Faecal egg count

## Abstract

Gastrointestinal nematodes are ubiquitous parasites of grazing equines with *Parascaris* spp., and strongyles being the most relevant ones regarding the prevalence and potential disease severity. Despite their importance, epidemiological data regarding the presence and egg-shedding intensities of these parasites are scarce. Data from 1067 horse samples collected on German horse farms initially to compare diagnostic methods were used for epidemiological analyses. Due to its higher sensitivity, presence/absence data were based on a combined sedimentation/flotation technique while faecal egg counts were based on Mini-FLOTAC. For strongyles, 46.5% of the samples were positive and the median egg-shedding intensity was 40 (range 5–2590). In multivariate analyses, prevalence and egg-shedding intensity were significantly influenced by season, age group and sample type. The drug used for the last treatment and the number of foals on the yard only affected prevalence while the number of horses on the yard and sex were only significant for egg-shedding intensity. For *Parascaris* spp., a prevalence of 4.6% and a median egg-shedding intensity of 0 (range 5–905) were observed. In multivariate analyses, the age group, the time since the last anthelmintic treatment, presence and number of foals had significant effects on ascarid prevalence whereas egg-shedding intensity was significantly influenced by age group and season only. *Parascaris* occurred only on yards with foals, but with an increasing number of foals, *Parascaris* egg-shedding intensity decreased. Prevalence and egg-shedding intensity were influenced by different but partially overlapping variables for *Parascaris* and strongyles.

**Supplementary Information:**

The online version contains supplementary material available at 10.1007/s00436-022-07765-4.

## Introduction

Like all grazing animals, horses are infected with a diverse number of pasture-borne gastrointestinal parasites among which the parasitic nematodes are the most important (Kaplan 2002; Matthews 2014; von Samson-Himmelstjerna 2012; Nielsen et al. 2010a). The reproductive stages of many of these parasites are shed in the faeces of horses, but the actual parasites and their frequencies that can be found in horses are influenced by environmental, host-specific and management factors (Corning 2009; von Samson-Himmelstjerna 2012; Nielsen et al. 2014b; von Samson-Himmelstjerna et al. 2009; Zanet et al. 2021). The environmental factors include abiotic and biotic variables such as humidity, temperature, soil type and plant communities on pasture while the most important management factors include treatment frequencies, the anthelmintic that was used and the access to pasture. The presence or absence of anthelmintic resistance is also related to management aspects. The most important host-specific factor is the age of the host but other factors such as sex, breed and general health (e.g. co-infections) may also be of relevance (Nielsen et al. 2010b, 2014b; Relf et al. 2013). The impact of environmental factors on the prevalence and abundance of gastrointestinal nematodes depends on the life cycle of the parasites, since different environmental stages differ in their susceptibility to effects such as evaporation and elevated temperature. In horses, the most frequently observed parasites in faecal samples belong to the groups of the strongyles and the ascarides (von Samson-Himmelstjerna 2012; Reinemeyer and Nielsen 2009) that exhibit strong differences in their life cycles (Nielsen et al. 2010b; Rehbein et al. 2013).

Strongyle nematodes of horses include the Strongylinae with the highly pathogenic *Strongylus* species and a large number of species (> 50) belonging to the Cyathostominae. These parasites almost always occur as coinfection of multiple species and up to 29 have already been identified in a single host individual (Bredtmann et al. 2017a; Johnson and Biddle 2021; Bucknell et al. 1995). Identification of these species is impossible using eggs, and only limited differentiation is possible based on third-stage larvae (L3). All these parasites share a similar non-parasitic phase of their life cycle with the development of infective third-stage larvae from eggs in the environment, which depends on many exogenic factors such as temperature, humidity and the type of ground the horses are kept on (e.g. amount of grass available, soil type, etc.). Infection occurs through oral uptake of L3. Although young horses are expected to be more susceptible to strongyles, these parasites frequently occur in all age groups.

In contrast to strongyles, the ascarids of equines (*Parascaris* spp.) undergo development to L3 in ovo and are well protected from many environmental stressors by their thick egg shell (Clayton and Duncan 1979b; Reinemeyer 2009; Clayton 1986; Lyons et al. 2011). Under suitable conditions such as in humid earth, ascarid eggs can survive for several years (Nielsen et al. 2019; Nielsen 2016; Reinemeyer 2012, 2009). The infection with *Parascaris* spp. occurs by oral uptake of eggs containing infective L3 (Reinemeyer 2009; Clayton and Duncan 1979b), and the parasites are highly pathogenic in foals and yearlings but are rarely found in older horses (Fritzen et al. 2010; Southwood et al. 1998; Rehbein et al. 2013; von Samson-Himmelstjerna et al. 2007; Hautala et al. 2019; Clayton and Duncan 1979a; von Samson-Himmelstjerna 2012).

Data about the prevalence and intensity of strongyle and ascarid egg shedding in Germany are scarce, and risk factor analyses are often based on a limited number of samples (Nielsen et al. 2014b, 2014a; Raue et al. 2017). Using a recently published dataset that was employed to compare different diagnostic methods for equine faecal samples (Boelow et al. 2022) and for which questionnaire data regarding the distribution of potential risk factors were available, the present study aims to identify risk factors for the presence of strongyle and ascarid eggs in equine faecal samples but also for egg-shedding intensity. In this study, all samples were analysed in parallel using combined sedimentation/flotation, Mini-FLOTAC and FECPAK^G2^. Since sedimentation/flotation was found to be the most sensitive of these methods (Boelow et al. 2022), data from this method were used to analyse data on the presence of parasites. Mini-FLOTAC was found to be the superior quantitative method in comparison to FECPAK^G2^ (Boelow et al. 2022), and thus, for statistical analysis of faecal egg count (FEC) data, the Mini-FLOTAC results were chosen.

## Materials and methods

### Faecal samples

All samples that were included were collected between 11/09/2017 and 13/08/2018 and originated from German horse farms. Samples were either sent to the diagnostics section of the Institute for Parasitology and Tropical Veterinary Medicine (*n* = 426) or were sent upon personal invitation specifically for the present study by horse owners (*n* = 469). Some of the samples (*l* = 172) have previously been used in a study by Jürgenschellert et al. (Jürgenschellert et al. 2020) and were reexamined for the present study. Since all samples were included in the method comparison study, only samples for which at least 35 g of faeces were available were included. For all samples, the answers to the standard questionnaire for diagnostic samples of the Institute for Parasitology and Tropical Veterinary Medicine (Supplementary Text S1) were available. Samples were refrigerated at 4–6 °C upon arrival and were analysed at the latest ten days after arrival.

### Combined sedimentation/flotation

For the combined sedimentation/flotation method, 15 g of faeces were resuspended in 40 ml of tap water as described previously (Boelow et al. 2022). Briefly, the suspension was filtered through an 800-µm-mesh-size sieve and centrifuged at 400 × g for 10 min. The pellet was resuspended in saturated sucrose solution (specific density 1.26) and centrifuged at 200 × g for 10 min before a horizontal wire loop was used to transfer three drops to a glass slide. For each egg type the results were categorised as negative if no eggs were found, + for 1–10 eggs, +  + for 11–40 eggs, +  +  + for 41 to 200 eggs and +  +  +  + for more than 200 eggs. For all analyses on the prevalence of egg shedding in the sample population, only data from sedimentation/flotation were used since this diagnostic approach yielded the highest number of positive samples and was therefore considered to be the most sensitive (Boelow et al. 2022).

### Mini-FLOTAC

Mini-FLOTAC was conducted as described recently (Boelow et al. 2022) using 5 g of faecal sample and 45 ml of saturated NaCl flotation solution (specific density 1.2) and counting all eggs in both counting chambers of the device. In order to calculate the number of eggs per gramme faeces, raw egg counts were multiplied by 5. For all analyses regarding egg-shedding intensity, results of the Mini-FLOTAC methods were used.

### Statistical analyses

Descriptive statistics of data (means, quartiles) were calculated in R version 4.1.1 using RStudio 1.4. and applying the summary function. For proportions, 95% confidence intervals (95% CIs) were calculated using the binom.wilson from the Epitools package. To determine if different levels of the same variable were observed with significantly different frequencies, binomial (binom.test) and multinomial (multinomial.test from the EMT 1.2 package) tests were conducted. Differences between proportions were analysed for significance using a mid-*p* exact test as implemented in the tab2by2.test function in the Epitools 0.5–10.1 package. Odds ratios were calculated using the exact mid-*p* method using oddsratio.midp from the same package. In a few cases, calculation with the exact mip-*p* method was impossible and exact Fisher’s method was applied using oddsratio.fisher from the same package. Distributions of quantitative variables between groups were compared using an unpaired Wilcoxon rank-sum test (wilcox.test) to compare two groups. For a comparison of more than two groups, a Kruskal–Wallis test (kruskal.test) was conducted followed by pairwise comparisons of all groups using Conover’s test (kwallPairsConoverTest from the PMCMRplus 1.9.3 package). Spearman correlations between two metric variables were calculated using the cor.test function from basic R. Effects of continuous and count variables on the prevalence of egg shedding were analysed using logistic regression analysis with a single explanatory variable. For this purpose, the glm function was used, and the family argument was set to “binomial”. Odds ratios and their 95% CIs were calculated from parameter estimates and standard errors by applying the confint function for the 95% CIs followed by exponentiation of estimates.

Multivariate analyses were conducted using the glm() function. For prevalence data, logistic regression analysis was used as described above but including all available variables in the initial model. This model was stepwise optimised by eliminating variables to decrease the Akaike information criterion (AIC). For the final model, Tjur’s D and Nagelkerke’s pseudo R^2^ were calculated using r2_tjur and r2_nagelkerke from the performance 0.4.4 package.

Regression models for egg-shedding intensity (egg counts in positive animals) were calculated using zero-truncated negative binomial regression using the vglm function from the VGAM package 1.1–6. Again, the initial model included all available variables and was stepwise reduced to optimise the AIC. For the final model, the Nagelkerke’s pseudo *R*^2^ was calculated using the PseudoR2 function implemented in the DescTools 0.99–45 package. Rate ratios were calculated as described above for odds ratios.

## Results

### Study population characteristics and potential explanatory variables

Table 1 summarises the information that was available for the 1067 horse samples included in the study. These horses came in total from 113 different sources with most of the sources representing yards but there were also a few equine clinics among the sources that sent-in samples of horses coming from different yards. The majority of the samples (64.9%) came from Brandenburg, the federal state surrounding Berlin, while 9.8% of the samples originated from Berlin. North Rhine-Westphalia, Lower Saxony and Bavaria contributed to 8.2%, 8.1% and 6.5% of the samples while only a few samples came from other federal states. With 23.0%, the proportion of foals in the study population was high. There were significantly more male horses in the study population (53.2%) than females (Table 1). No discrimination was made between stallions and geldings since according to the questionnaire responses only 178 of the 549 males had been neutered, which would correspond to a proportion of 67.6% of stallions among the males. This appeared to be unrealistic and data regarding the castration of male horses were therefore considered unreliable.

**Table 1:** Characteristics of the study population (*n* = 1067) included in the present study

Parameter	Categories	*N*^a^	*n*^b^	Frequency (%)^c^	Median (range)^d^	Significance^e^
Shipping time (days)		1066	n.a		1 (0–22)	
Sample type	Individual	1067	995	93.26		< 0.001
	Composite^f^		72	6.74		
Sex	Male	1031	549	53.25		0.002
	Female		482	46.75		
No. horses/yard		955	n.a		52 (1–200)	
No. foals/yard		737	n.a		2 (0–60)	
Presence of foals	Yes	737	428	58.07		
Age group	Foals (< 1 year)	1037	239	23.04		< 0.001
	Yearlings (1–4 years)		79	7.62		
	Adults (> 4 years)		719	69.33		
Last treatment^g^	Pyrantel	833	121	14.53		< 0.001
	Ivermectin		469	56.30		
	Moxidectin		169	20.29		
	Doramectin		5	0.60		
	Ivermectin + pyrantel		13	1.56		
	Fenbendazole		52	6.24		
	Homeopathic (Abronatum)		4	0.48		
Time since last treatment^g^	< 8 weeks	967	237	24.79		< 0.001
	≥ 8 weeks		730	76.36		
Season	Spring	1067	406	38.05		
	Summer		151	14.15		
	Autumn		225	21.09		
	Winter		285	26.71		
State of origin	Brandenburg	1067	693	64.9		
	Berlin		105	9.84		
	North Rhine-Westphalia		87	8.15		
	Lower Saxony		86	8.06		
	Bavaria		69	6.47		
	Baden-Württemberg		14	1.31		
	Mecklenburg-Western Pomerania		6	0.56		
	Schleswig–Holstein		6	0.56		
	Hesse		1	0.09		

^a^*N*, Total number of samples for which this information was available

^b^*n*, Number of samples belonging to this category, only for categorial data

^c^Only for categorial variables

^d^Only for continuous variables

^e^Differences in frequency of categories in binomial (two categories) or multinominal (at least three categories) test

^f^Composite samples from the same horse collected over several days and pooled

^g^Only nematocidal drugs included

*n.a.*, not available

For 76.4% of the horses, the last treatment was more than 8 weeks ago (Table 1). The most frequently used anthelmintic that was used for the last treatment was ivermectin (56.3%), followed by moxidectin (20.3%), pyrantel (14.5%) and fenbendazole (6.1%). Noteworthy, five horses, all from the same yard, received doramectin as last treatment, a drug that is not licenced for treating horses in Germany (Table 1) but was also used, as in past studies (Fritzen et al. 2010; Matthee 2003), probably for cost reasons by some horse owners.

The vast majority of the samples were individual samples (93.3%) while the rest was collected from an individual horse but pooled from consecutive days (Table 1). Since samples were either brought directly to the Institute for Parasitology and Tropical Veterinary Medicine or sent by parcel services, the shipping time was also considered a relevant variable. The median shipping time was 1 day (range 0–22 days) but 79.1% of the samples had a shipping time of two days or less.

### Prevalences of helminth species

Data regarding the prevalence of helminth species observed in this data set have already been published recently (Boelow et al. 2022) but are summarised here again in Table 2. For strongyle and *Parascaris* spp. enough samples were positive to perform risk factor analyses.

**Table 2:** Prevalence and 95% confidence intervals (95% CIs) for helminth species detected using sedimentation/flotation in the 1067 samples included in the study

Species	*n*^a^	Prevalence (%)	95% CI
Individual infections^b^			
Strongyles	496	46.5	43.5–49.5
*Parascaris* spp.	49	4.6	3.5–6.0
*Strongyloides westeri*	6	0.6	0.3–1.2
*Oxyuris equi*	6	0.6	0.3–1.2
Anoplocephalidae	29	2.7	1.9–3.9
Co-infections^c^			
Strongyles/*Parascaris* spp.	23	2.16	0.01–0.03
Strongyles/*S. westeri*	5	0.47	0.001–0.009
Strongyles/*O. equi*	3	0.28	0–0.01
Strongyles/Anaplocephalidae	20	1.87	0.01–0.03
Strongyles */Parascaris* spp./*S. westeri*	1	0.09	0–0.003
Strongyles*/Parascaris* spp./*O. equi*	1	0.09	0–0.003

^a^*n*, number of positive samples

^b^Data here include also animals that were infected with more than one parasite

^c^Co-infection data list all animals only in the category naming all parasites found in the host

### Strongyle egg shedding

#### Bivariate data analyses for prevalence of strongyle egg shedding according to sedimentation/flotation data

For categorial variables, mid-*p* exact tests were conducted to determine if the frequency of horses positive for strongyle egg shedding was different between the levels of the variable. If a variable had more than two levels, mid-*p* exact tests were conducted for each combination of the different levels. Results of these analyses are presented in Table 3.

**Table 3:** Comparison of prevalence for strongyle egg shedding between levels of categorial variables in bivariate analyses

Variable	Level	Number pos./total^a^	Prevalence (95% CI^b^) [%]	OR^c^ (95% CI^b^)	Significant to other levels^d^
Sex	Male	247/549^e^	45.0 (40.9–49.2)	1	
	Female	228/482	47.3 (42.9–51.8)	1.10 (0.86–1.40)	
Presence of foals	Yes	230/428	53.7 (49.0–58.4)	1	No
	No	129/309	41.7 (36.4–47.3)	0.62 (0.46–0.83)	Yes
Age group	Foals (< 1 year)	117/239	49.0 (42.3–55.3)	1.31 (0.98–1.76)	Yearlings
	Yearlings (1–4 years)	58/79	73.4 (62.8–81.9)	3.75 (2.26–6.45)	Foals, adults
	Adults (> 4 years)	304/719	42.3 (38.7–45.9)	1	Yearlings
Last treatment	Pyrantel	80/121	66.1 (57.3–73.9)	1	IVM, MOX, FEN
	Ivermectin	238/469	50.7 (46.2–55.2)	0.52 (0.35–0.80)	PYR, MOX, DOR
	Moxidectin	33/169	19.5 (14.2–26.2)	0.13 (0.07–0.21)	PYR, IVM, DOR, FEN, IVM/PYR
	Doramectin	5/5	100 (56.6–100)	Not calculable	IVM, MOX, FEN
	Fenbendazole	20/52	38.5 (26.5–52.0)	0.32 (0.15–0.66)	PYR, MOX, DOR, IVM/PYR
	Ivermectin/pyrantel	10/13	76.9 (49.7–91.8)	1.70 (0.41–10.15)	MOX, FEN
Time since last treatment^f^	< 8 weeks	105/237	44.3 (38.1–50.7)	1	
	≥ 8 weeks	334/730	45.8 (42.2–49.4)	1.06 (0.79–1.43)	n.s
Season	Spring	124/406	30.5 (26.3–35.2)	1	Su, Au, Wi
	Summer	80/151	53.0 (45.0–60.8)	2.56 (1.74–3.76)	Sp, Au
	Autumn	159/225	70.7 (64.4–76.2)	5.46 (3.84–7.83)	Sp, Su, Wi
	Winter	133/285	46.7 (41.0–52.5)	1.99 (1.45–2.73)	Sp, Au
State of origin	Brandenburg	320/693	46.2 (42.5–49.9)	2.37 (1.46–3.99)	BE, NW, BY
	Berlin	47/105	44.8 (35.6–54.3)	2.14 (1.49–3.08)	BB, NW
	North Rhine-Westphalia	23/87	26.4 (18.3–36.6)	1	BE, BB, NI, BY
	Lower Saxony	49/86	57.0 (46.4–66.9)	3.64 (1.93–7.03)	NW
	Bavaria	43/69	61.3 (50.5–72.8)	4.54 (2.32–9.14)	BE, NW
	Baden-Württemberg	6/14	42.9 (21.4–67.4)	n.a.^g^	n.a.^g^
	Mecklenburg-Western Pomerania	6/6	100 (61.0–100)	n.a.^g^	n.a.^g^
	Schleswig–Holstein	2/6	33.3 (9.7–70.0)	n.a.^g^	n.a.^g^
	Hesse	0/1	0 (0–79.3)	n.a.^g^	n.a.^g^
Sample type	Individual	471/995	47.1 (44.1–50.4)	1	Composite
	Composite	25/72	34.7 (24.8–46.3)	0.59 (0.35–0.97)	Individual

^a^Numbers of positive/total horse samples

^b^*CI*, confidence interval

^c^*OR*, odds ratio obtained in the mid-*p* exact test

^d^Significant difference (*p* < 0.05) in a mid-*p* exact test against the named levels of the same variable without *p* value adjustment for multiple testing. *DOR*, doramectin; *FEN*, fenbedazole; *IVM*, ivermectin; *PYR*, pyrantel; *BB*, Brandenburg; *BE*, Berlin; *BY*, Bavaria; *NI*, Lower Saxony; *NW*, North Rhine-Westphalia

^e^Including neutered animals

^f^Odds ratios were calculated using Fisher’s exact test since the mid-*p* exact test lead to numerical problems and errors

^g^Excluded due to small number of animals

*n.a.*, not available; *n.s.*, not significant

The sex of the horses had no significant effect on the prevalence of strongyle egg shedding. Regarding the age groups, yearlings had a significantly higher prevalence than foals and adults. Although prevalence in foals was higher than in adults, this difference was not significant (Table 3). The effect of the drug used for the last treatment was significant. After treatment with moxidectin, the frequency of positive horses was the lowest (19.5%) followed by fenbendazole (38.5%), ivermectin (50.7%), the combination of ivermectin/pyrantel (76.9%) and pyrantel (82.3%). Five horses, all from the same yard, had been treated with doramectin, which is not licenced for the treatment of horses, and they were all positive for strongyle eggs. For information about which differences between individual drugs were significant, see Table 3. Whether the last treatment was more than eight weeks ago or not, had no significant influence on the frequency of positive horses (Table 3).

The presence of foals on the yard led to a significantly higher frequency of horses positive for strongyle-type eggs. The state in which the yard was located was also relevant with the highest prevalence observed in Berlin followed by Bavaria, Lower Saxony, Brandenburg and North Rhine-Westfalia (some states with low numbers of animals in the study population were excluded from the analysis) (see Table 3).

The highest number of positive samples was detected in autumn followed by summer, winter and spring. All comparisons were significant (Table 3).

Finally, individual and composite samples (from the same horse from successive days) were compared. Unexpectedly, composite samples were significantly less positive than individual samples (Table 3).

For continuous explanatory variables, logistic regression analyses were performed. The variables “number of horses” and “number of foals per farm” and the “shipping time” before the sample arrived in the laboratory were considered. A significant but small protective effect of an increasing number of horses on the yard was observed while numbers of foals and shipping time had no significant effects (Supplementary Table S1).

#### Bivariate analysis of intensity of strongyle egg shedding according to Mini-FLOTAC data

The distribution of strongyle egg counts is described in Table 4. Since the 25% quantile was almost always 0 and even for the vast majority of variables the medians were 0, the table provides the median, the 75% quantile and the maximum as well as the mean. On farms with foals, the eggs per gramme faeces (EPG) were significantly higher than on farms without foals. The age group also had a significant effect on the EPG, with the yearlings showing higher EPGs than foals and adults. The last treatment with moxidectin resulted in significantly lower EPGs than the treatment with any other drug. However, several other comparisons between drugs also found significant differences (see Table 4 for details). The season and the geographical origin also had a significant influence on egg count characteristics with many of the individual comparisons showing significant differences (Table 4). Finally, composite samples had significantly lower EPGs than individual samples. No effect was observed for the sex and the time since the last treatment with an anthelmintic. To investigate the effect of continuous variables on FECs, Spearman correlations between abundance and intensity and these variables were calculated. Regarding abundance, a high number of foals had a significant positive effect on the EPG, whereas an increasing number of horses and longer shipping times resulted in lower EPGs (Supplementary Table S2). In contrast, significant effects on egg-shedding intensity were only observed for shipping time (Supplementary Table S2).

**Table 4:** Comparison of strongyle egg shedding abundance and intensity between levels of categorial variables

		Abundance (*N* = 1067)				Intensity (*N* = 436)			
Variable	Level	*n*	Mean	Median (75% quantile/maximum)	Significant to other levels^a^	*n*	Mean	Median (range)	Significant to other levels^a^
Sex	Male^b^	549^e^	87.8	0 (30/2150)	n.s	215	224.2	80 (5–2150)	n.s
	Female	482	102.0	0 (50/2590)	n.s	202	243.3	85 (5–2590)	n.s
Presence of foals	Yes	428	144.9	0 (100/2590)	No	108	189.3	62.5 (5–1440)	n.s
	No	309	66.2	0 (20/1440)	Yes	207	299.6	115 (5–2590)	n.s
Age group	Foals (< 1 year)	239	147.8	0 (95/2150)	Yearlings, adults	101	349.8	155 (5–2150)	Adults
	Yearlings (1–4 years)	79	251.1	65 (435/1590)	Foals, adults	56	354.3	175 (5–2590)	Adults
	Adults (> 4 years)	719	64.9	0 (20/1595)	Foals, yearlings	44	176.9	52.5 (5–1595)	Foals, yearlings
Last treatment	Pyrantel	121	116.6	15 (140/1060)	FEN, IVM, MOX	72	195.9	85.0 (5–1060)	n.s
	Ivermectin	469	130.9	0 (60/2590)	PYR, MOX, DOR	207	296.5	105 (5–2590)	n.s
	Moxidectin	169	38.4	0 (0/1595)	PYR, IVM, DOR, PYR/IVM	26	249.4	30.0 (5–1595)	n.s
	Doramectin	5	323.0	165 (385/840)	IVM, MOX, FEN	5	323.0	165 (80–849)	n.s
	Fenbendazole	52	48.0	0 (6.25/1325)	PYR, DOR	18	138.6	37.5 (5–1325)	n.s
	Ivermectin/pyrantel	13	162.7	10 (240/805)	MOX	9	235.0	135.0 (10–805)	n.s
Time since last treatment^f^	< 8 weeks	237	111.9	0 (25/1765)	n.s	89	298.0	95.0 (5–1765)	n.s
	≥ 8 weeks	730	96.6	0 (40/2590)	n.s	296	238.2	92.5 (5–2590)	n.s
Season	Spring	406	34. 5	0 (5/1125)	Su, Au, Wi	108	129.6	42.5 (5–1125)	Su, Au
	Summer	151	205.6	0 (175/2590)	Sp, Wi	142	233.8	82.5 (5–1650)	Sp, Au
	Autumn	225	147.5	15 (165/1650)	Sp, Wi	74	419.6	180.0 (5–2590)	Sp, So
	Winter	285	89.8	0 (30/2150)	Sp, So, Au	112	228.4	95.0 (5–2150)	So
State of origin	Brandenburg	693	99.1	0 (45/2590)	BY, NW	146	235.3	77.5 (5–2590)	n.s
	Berlin	105	77.0	0 (30/1595)	BY	38	216.6	90 (5–1595)	n.s
	North Rhine-Westphalia	87	71.8	0 (0/1125)	BB, NI, BY	18	346.9	212.5 (5–1125)	n.s
	Lower Saxony	86	90.8	5 (98.8/1030)	NW	44	177.4	97.5 (5–1030)	n.s
	Bavaria	69	217	50 (170/1650)	BB, BE, NW	39	284.1	95 (5–1650)	n.s
	Baden-Württemberg	14	88.2	0 (75/455)	n.a.^c^	4	308.8	340 (100–455)	n.a.^c^
	Mecklenburg-Western Pomerania	6	117.5	25 (28.8/615)	n.a.^c^	5	141.0	25 (10–615)	n.a.^c^
	Schleswig–Holstein	6	1.7	0 (3.8/5)	n.a.^c^	2	5	5 (5–5)	n.a.^c^
	Hesse	1	0	0 (0/0)	n.a.^c^	0	n.a	n.a	n.a
Sample type	Individual	995	102.6	0 (50/2590)		417	244.9	95 (5–2590)	Composite
	Composite	72	23.5	0 (5/870)		19	89.2	40 (5–870)	Individual
Total	n.a	1067	97,3	0 (40/2590)	n.a	436	238,12	40 (5–2590)	n.a

^a^Significant difference (*p* < 0.05) in a Wilcoxon signed-rank test (comparison between two states) or a Kruskal–Wallis test followed by a Conover-Iman post hoc test (three or more states) against the named levels of the same variable. *DOR*, doramectin; *FEN*, fenbedazol; *IVM*, ivermectin; *PYR*, pyrantel; *Sp*, spring; *Su*, summer; *Au*, autumn; *Wi*, winter; *BB*, Brandenburg; *BE*, Berlin; *BY*, Bavaria; *NI*, Lower Saxony; *NW*, North Rhine-Westphalia

^b^Including neutered animals

^c^Excluded due to small number of animals

*n.a.*, not available; *n.s.*, not significant

#### Multivariate analysis to identify risk factors for the shedding of strongyle eggs

**Fig. 1:**
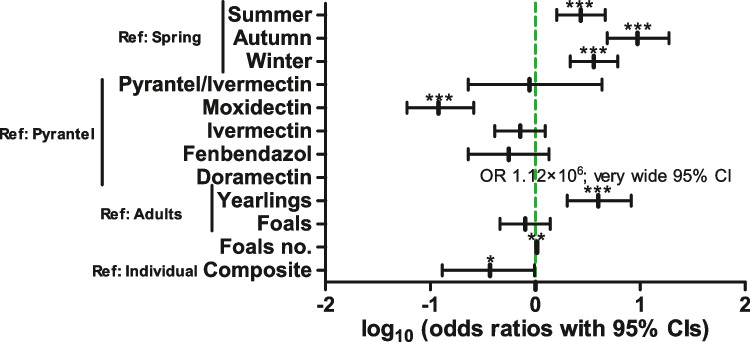
Forest plot showing odds ratios with 95% confidence intervals (CIs) for logistic regression analysis to identify risk factors for being positive for strongyle egg shedding. Samples were analysed by sedimentation/flotation. OR, odds ratio; Ref, reference level; ***p < 0.001; **p < 0.01; *p < 0.05 in *t* test

Multivariate analysis included all independent variables used in the bivariate analyses in the initial binomial regression model. The model was then optimised by stepwise elimination of variables aiming to improve (decrease) the AIC. Odds ratios of the final model are visualised in Fig. 1 while additional details of the model are summarised in Supplementary Table S3. There was a significant effect of the drug used for the last treatment with moxidectin resulting in significantly lower odds to be positive than the reference pyrantel. All other drugs did not significantly differ from pyrantel (Fig. 1). For doramectin, the odds ratio was very high, but the 95% CI was also very wide due to the very small number of horses treated with this drug and the facts that they were all positive and all came from the same farm leading to a correlation between explanatory variables on this farm. In spring, the odds to be positive were significantly lower than in any of the other seasons. Regarding the age of the animals, yearlings had significantly higher odds to be strongyle positive than adults, but there was no significant difference between adults and foals. Looking at the 95% CIs, the difference between yearlings and foals is obviously also significant. Despite that, the number of foals on the yard was associated with higher odds to be positive for strongyles. Finally, the sample type had a significant effect on the results with composite samples collected over several days having significantly lower odds to be positive than individual samples collected on a single day (Fig. 1, Supplementary Table S3).

#### Multivariate analysis to identify risk factors for the intensity of strongyle egg shedding

**Fig. 2:**
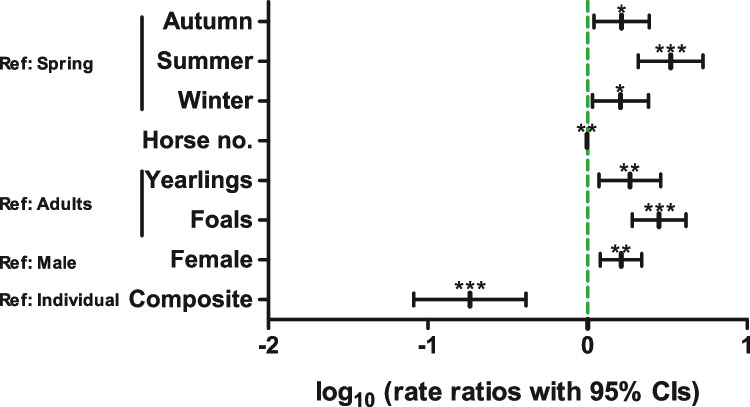
Forest plot showing rate ratios with 95% confidence intervals (CIs) for a zero-truncated negative binomial regression analysis on data of horses positive for strongyle egg shedding to identify risk factors for intensity of strongyle egg shedding. Eggs per gramme faeces were determined by Mini-FLOTAC. Ref., reference level; ***p < 0.001; **p < 0.01; *p < 0.05 in *t* test

To determine factors that influence egg-shedding intensity, only data from positive animals were included. Data were analysed using zero-truncated negative binomial regression. The final model is presented as a forest plot in Fig. 2 and in more detail in Supplementary Table S4. Variables such as season, age group and sample type that had a significant impact in the logistic regression model also had a significant influence on the intensity of egg shedding. While the effects of season and sample type were very similar in both models, this was only partially the case for the age group. Here, yearlings had both significantly higher odds to be positive than adults and higher egg-shedding intensity than adults. The situation for foals was different. Although foals had no higher odds to be positive for strongyle egg shedding, egg counts of positive foals were significantly higher than those of positive adult horses. The variables “anthelmintic used for last treatment” and “number of foals” significantly influenced the odds to be strongyle positive in faeces while they were not included in the final model for egg-shedding intensity. In contrast, the sex (with a higher rate ratio for females) and the number of horses on the yard had a significant influence on the egg-shedding intensity while they were not included in the final model for the presence/absence of strongyle eggs in the faeces.

### Parascaris spp. egg shedding

#### Bivariate data analyses for prevalence of Parascaris spp. egg shedding according to sedimentation/flotation data

The number of samples positive for *Parascaris* spp. was about tenfold lower than for strongyles (Table 2). Nevertheless, quite a number of significant effects could be identified as detailed in Table 5.

**Table 5:** Comparison of prevalence for *Parascaris* spp. egg shedding between levels of categorial variables

Variable	Level	Number pos./total^a^	Prevalence (95% CI) [%]^b^	OR (95% CI)^c^	Significant to other levels^d^
Sex^e^	Male	26/549	4.7 (3.3–6.8)	1	
	Female	20/482	4.1 (2.7–6.3)	0.87 (0.47–1.58)	
Presence of foals	Yes	47/428	11.0 (8.4–14.3)	1	No
	No	0/309	0 (0–1.2)	0.00 (0.00–0.07)^f^	Yes
Age group	Foals (< 1 year)	44/239	18.4% (14.0–23.8)^x^	1	Yearlings, adults
	Yearlings (1–4 years)	3/79	3.4 (1.3–10.6)	0.0006 (0.00008–0.000.0015)	Foals, adults
	Adults (> 4 years)	2/719	0.3 (0.1–1.0)	0.00 (0.00–0.00)	Foals, yearlings
Last treatment	Pyrantel	9/121	7.4 (4.0–13.5)	0.5 (0.2–1.7)^f^	IVM
	Ivermectin	11/469	2.3 (1.3–4.2)	0.2 (0.1–0.5)^f^	FEN, PYR
	Moxidectin	6/169	3.6 (1.6–7.5)	0.2 (0.1–0.9)^f^	FEN
	Doramectin	0/5	0 (0–43.4)	0 (0–8.6)^f^	n.s
	Fenbendazole	7/52	13.5 (6.7–25.3)	1^f^	IVM, MOX
	Ivermectin/pyrantel	0/13	0 (0–22.8)	0 (0–2.8)^f^	n.s
Time since last treatment^e^	< 8 weeks	19/237	8.0 (5.2–12.2)	1	≥ 8 weeks
	≥ 8 weeks	27/730	3.7 (2.6–5.3)	0.42 (0.23–0.79)	< 8 weeks
Season	Spring	19/406	4.7 (3.0–7.2)	1.75 (0.64–6.28)	n.s
	Summer	4/151	2.6 (1.0–6.6)	1	n.s
	Autumn	8/225	3.6 (1.8–6.9)	1.33 (0.40–5.24)	n.s
	Winter	18/285	6.3 (4.0–9.8)	2.40 (0.87–8.66)	n.s
State of origin	Brandenburg	33/693	4.8 (3.4–6.6)	0.55 (0.25–1.42)	n.s
	Berlin	1/105	1.0 (0.1–5.2)	0.12 (0.00–0.72)	NW, NI
	North Rhine-Westphalia	7/87	8.0 (4.0–15.7)	0.99 (0.32–3.07)	BE
	Lower Saxony	7/86	8.1 (4.0–15.7)	1	BE
	Bavaria	1/69	1.4 (0.2–7.8)	0.19 (0.01–1.12)	n.s
	Baden-Württemberg	0/14	0 (0–21.5)	n.a.^g^	n.a.^g^
	Mecklenburg-Western Pomerania	0/6	0 (0–39.0)	n.a.^g^	n.a.^g^
	Schleswig–Holstein	0/6	0 (0–39.0)	n.a.^g^	n.a.^g^
	Hesse	0/1	0 (0–79.3)	n.a.^g^	n.a.^g^
Sample type	Individual	48/995	4.8 (3.7–6.3)	1	n.s
	Composite	1/72	1.4 (0.2–7.5)	0.32 (0.01–1.46)	n.s

^a^Numbers of positive/total horse samples

^b^*CI*, confidence interval

^c^*OR*, odds ratio

^d^Including neutered animals

^e^Significant difference (*p* < 0.05) in a mid-*p* exact test against the named levels of the same variable. *DOR*, doramectin; *FEN*, fenbendazole; *IVM*, ivermectin; *PYR*, pyrantel; *Sp*, spring, *Su*, summer; *Au*, autumn; *Wi*, winter; *BB*, Brandenburg; *BE*, Berlin; *BY*, Bavaria; *NI*, Lower Saxony; *NW*, North Rhine-Westphalia

^f^Odds ratios were calculated using Fisher’s exact test since the mid-p exact test lead to numerical problems and errors

^g^Excluded due to small number of animals

*n.a.*, not available; *n.s.*, not significant

As observed for strongyles, no significant difference between male and female horses was observed (Table 5). The age group had a clear effect on the frequency of positive horses and in contrast to the strongyles there was a clear age effect with by far the highest prevalence in the foal group (18.4%) followed by yearlings (3.4%) and adults (0.3%). Regarding the effect of the drug that was used for the last treatment, no *Parascaris* spp. positive samples were observed for the combination of ivermectin/pyrantel and for the not licenced doramectin, but the number of animals treated with these drugs was very small for both drugs. Among the more frequently used drugs, prevalence was lowest among the animals treated with ivermectin followed by moxidectin, pyrantel and fenbendazole. Numbers of positive animals for all of the treatment groups were small (maximum 11) which shows that all conclusions drawn from these should be considered carefully. Remarkably, the prevalence of *Parascaris* spp. egg shedding was significantly higher (almost twice as high) in horses for which the last treatment was less than eight weeks ago compared to animals treated at least eight weeks ago (Table 5).

The presence of foals was clearly associated with a higher prevalence of *Parascaris* spp. In fact, this parasite was exclusively observed on these farms (Table 5). Regarding the geographic origin of samples, only a few significant effects were observed, which is also explainable by the small number of positive horses. Only Berlin was found to have a significantly lower prevalence than Lower Saxony and North Rhine-Westfalia while all other differences were not significant (Table 5).

Neither the season when a sample was collected nor whether it was an individual or a composite sample had any significant effect on the frequency of *Parascaris* positive samples.

Fitting of bivariate logistic regression models for the variables “number of horses” and “foals per yard and shipping time”, as performed for strongyle egg shedding, was not possible due to the small number of positive horses.

#### Bivariate analysis of abundance and intensity of Parascaris spp. egg shedding according to Mini-FLOTAC data

Only a small number of independent variables had a significant effect on *Parascaris* spp. FECs. In terms of abundance, farms with foals had significantly higher EPGs since the parasite did not occur on any farm without foals (Table 6). Accordingly, there were also significant differences between the age groups with significantly higher FECs in foals than yearlings and adults. Although the parasite was not found in adult horses at all, the differences between yearlings and adults were not significant since in yearlings also only three positive horses with a maximum EPG of 25 were found. Regarding the last treatment, moxidectin- and ivermectin-treated animals had significantly lower EPGs than animals treated with fenbendazole (Table 6). Moreover, the EPG was significantly higher in horses for which the last treatment was less than eight weeks ago than for animals with the last anthelmintic treatment at least eight weeks ago. Neither sex, season, state of origin nor sample type had an effect on the intensity of *Parascaris* spp. egg shedding (Table 6).

**Table 6:** Comparison of *Parascaris* spp. egg shedding abundance and intensity between levels of categorial variables

Variable	Level	Abundance (*N* = 1067))			Significant to other levels^a^	Intensity (*N* = 43)			Significant to other levels^a^
		*n*	Mean	Median (75% quantile/maximum)		*n*	Mean	Median (range)	
Sex^b^	Male	549	3.97	0 (0/695)	n.s	24	90.8	22.5 (5–695)	n.s
	Female	482	3.3	0 (0/680)	n.s	15	106.3	40 (5–680)	n.s
Presence of foals	Yes	428	12.4	0 (0/905)	No	41	129.6	40 (5–905)	n.s
	No	309	0	0 (0/0)	Yes	2	185	185 (10–360)	n.s
Age group	Foals (< 1 year)	239	23.5	0 (0/905)	Yearlings, adults	39	143.7	40 (5–905)	n.s
	Yearlings (1–4 years)	79	0.5	0 (0/25)	Foals	3	13.3	10 (5–25)	n.s
	Adults (> 4 years)	719	0.1	0 (0/40)	Foals	1	40	40 (40)	n.s
Last treatment	Pyrantel	121	11.0	0 (0/905)		8	166.9	60 (5–905)	n.s
	Ivermectin	469	1.6	0 (0/200)	FEN	8	91.9	55 (10–200)	n.s
	Moxidectin	169	3.7	0 (0/230)	FEN	7	90	40 (10–230)	n.s
	Doramectin	5	0	0 (0/0)		0	n.a	n.a	n.s
	Fenbendazole	52	17.2	0 (0/700)	IVM, MOX	8	111.9	17.5 (10–700)	n.s
	Ivermectin/pyrantel	13	0	0 (0/0)		0	n.a	n.a	n.s
Time since last treatment^f^	< 8 weeks	237	13.0	0 (0/905)	≥ 8 weeks	19	146.7	40 (10–905)	n.s
	≥ 8 weeks	730	3	0 (0/695)	< 8 weeks	21	115.3	25 (5–695)	n.s
Season	Spring	406	0.8	0 (0/105)	n.s	15	22.3	10 (5–105)	Su, Au, Wi
	Summer	151	12.3	0 (0/905)	n.s	5	370	115 (25–905)	Sp
	Autumn	225	8.7	0 (0/695)	n.s	6	325	265 (5–695)	Sp
	Winter	285	5.4	0 (0/230)	n.s	17	91.2	45 (10–230)	Sp
State of origin	Brandenburg	693	4.6	0 (0/695)	n.s	31	101.8	25 (5–695)	n.s
	Berlin	105	3.4	0 (0/360)	n.s	1	360	360 (360)	n.s
	North Rhine-Westphalia	87	1.7	0 (0/75)	n.s	5	29	10 (5–75)	n.s
	Lower Saxony	86	23.5	0 (0/905)	n.s	6	337.5	115 (10–905)	n.s
	Bavaria	69	0	0 (0/0)	n.s	0	n.a	n.a	n.a
	Baden-Württemberg	14	0	0 (0/0)	n.a.^c^	0	n.a	n.a	n.a
	Mecklenburg-Western Pomerania	6	0	0 (0/0)	n.a.^c^	0	n.a	n.a	n.a
	Schleswig–Holstein	6	0	0 (0/0)	n.a.^c^	0	n.a	n.a	n.a
	Hesse	1	0	0 (0/0)	n.a.^c^	0	n.a	n.a	n.a
Sample type	Individual	995	5.5	0 (0/905)	n.s	42	130.8	32.5 (5–905)	n.s
	Composite	72	2.6	0 (0/190)	n.s	1	190	190 (190)	n.s
Total	n.a	1067	5.3	0 (0/905)	n.a	43/1067	132.2	0 (5–905)	n.a

^a^Significant difference (*p* < 0.05) in a Wilcoxon signed-rank test (comparison between two states) or a Kruskal–Wallis test followed by a Conover-Iman post hoc test (three or more states) against the named levels of the same variable. *DOR*, doramectin; *FEN*, fenbendazole; *IVM*, ivermectin; *PYR*, pyrantel; *BB*, Brandenburg; *BE*, Berlin; *BY*, Bavaria; *NI*, Lower Saxony; *NW*, North Rhine-Westphalia

^b^Including neutered animals

^c^Excluded due to small number of animals

*n.a.*, not available; *n.s.*, not significant

Since the number of positive horses was only 43, the number of significant differences was even smaller for the intensity of egg shedding measured in EPG. In fact, the only variable with influence in the binomial analyses was the season, with FEC being significantly lower in Spring compared to all other seasons (Table 6).

For continuous independent variables, Spearman correlations were calculated. The number of foals had a significant positive effect on the abundance of *Parascaris* spp. FECs (Supplementary Table S5). In contrast, there was no significant correlation between the number of horses or shipping time and the *Parascaris* spp. EPG. None of the variables affected the intensity of egg shedding significantly (Supplementary Table S5).

#### Multivariate analysis to identify risk factors for the presence of Parascaris spp. eggs

**Fig. 3:**
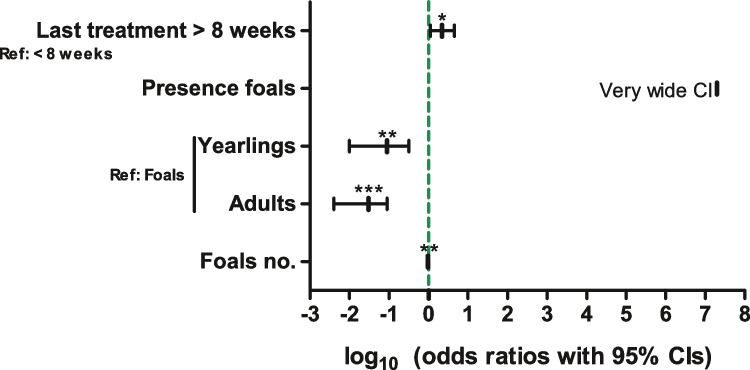
Forest plot showing odds ratios with 95% confidence intervals (CIs) for logistic regression analysis to identify risk factors for being positive for *Parascaris* spp. egg shedding. Samples were analysed by sedimentation/flotation. OR, odds ratio; Ref., reference level; ***p < 0.001; **p < 0.01; *p < 0.05 in *t* test

In a multivariate analysis, only a few variables could be identified as risk or protective factors for the odds to be positive for *Parascaris* spp. as summarised in Fig. 3 and Supplementary Table S6. If at least eight weeks had passed since the last treatment, there was a significant increase in the odds to be positive. Moreover, adults and yearlings had significantly lower odds to be positive than foals. An increasing number of foals on a farm was slightly protective whereas the presence of foals on a farm led to a very high OR of 1.9 × 10^7^, but this effect was not significant due to a very wide 95% CI.

#### Multivariate analysis to identify risk factors for the intensity of Parascaris spp. egg shedding

**Fig. 4:**
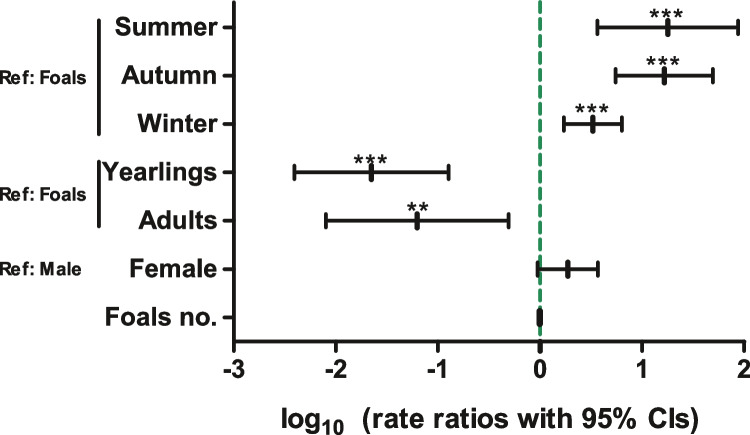
Forest plot showing rate ratios with 95% confidence intervals (CIs) for zero-truncated negative binomial regression analysis to identify risk factors for intensity of *Parascaris* spp. egg shedding. Eggs per gramme faeces were determined by Mini-FLOTAC. RR, rate ratio; Ref., reference level; ***p < 0.001; **p < 0.01 in *t* test

Results of the zero-truncated negative binomial analysis of EPG data for *Parascaris* spp. are shown in Fig. 4 and Supplementary Table S7. As already observed in the bivariate analysis, a significant higher egg-shedding rate ratio was observed for summer, autumn and winter in comparison to spring. In contrast to the bivariate analysis results and despite the fact that only three and one animal were positive in the age groups of yearlings and adults respectively, these age groups were associated with significantly lower egg counts (Fig. 4). Other variables in the final model that improved the AIC but had no significant effect were the number of foals on the yard, which was associated with a lower egg-shedding intensity and the sex with a tendency of higher egg-shedding intensity in females than males.

## Discussion

The epidemiology of parasites of domestic horses depends on (i) host and parasite interactions leading, e.g. to a preference of certain host age groups or genotypes and (ii) environmental factors that in particular affect the survival of off-host stages of parasites but also (iii) on human/management factors such as pasture hygiene, treatment schemes and anthelmintic drug used. While human activity directly affects parasite prevalence and abundance, parasite populations also show considerable plasticity and respond by adaptation to human intervention for instance with the evolution of resistance to anthelmintics to mention just the best studied among these phenomena. This scenario predicts dynamic changes in abundance, intensity and prevalence of parasites in host populations depending on changes in environmental conditions, husbandry practices and available anthelmintics. Therefore, epidemiological data require frequent updates to make sure that recommendations for optimal husbandry are up to date.

Studies aiming to provide estimates for the prevalence and intensity of equine parasites are almost never conducted using a truly representative study design concerning, e.g. aspects of sample size or sample selection. To reduce these biases, there is an Australian metaanalysis that summarises epidemiological data from 51 different studies on gastrointestinal parasites in horses (Saeed et al. 2019). This study certainly gives a good cross-section, but again, it is only as good as the sample selection of the individual studies and could be biased in a certain direction. Since the study is limited to Australia, the results cannot be applied to other countries or continents. A more representative study, however, is Bellaw and Nielsen (2020), which summarises 37 study results from over 45 years in different countries.

For example, cross-sectional studies typically suffer from non-random selection of participating farms and often also non-random selection of horses within a farm since individual horses are often owned by different people who decide according to individual preferences if their animal should be sampled or not. Longitudinal studies are well suited to investigate seasonal or age-dependent factors but typically are conducted on only a few study sites in parallel and might therefore be biased. For a certain region such as Germany, the number of cross-sectional (Ertelt et al. 2015; Hinney et al. 2011; Schneider et al. 2014) and longitudinal (Rehbein et al. 2013; Scheuerle et al. 2016) studies that have been conducted in the last two decades is usually quite limited. Another source of relevant data can come from diagnostic samples (Raue et al. 2017). Such data are also subject to certain types of bias such as higher frequency of samples derived from clinically conspicuous animals, non-representative age structure and dependency on the attitude of owners regarding the importance of regular diagnostic checkups. In comparison to cross-sectional and longitudinal studies that involve visits of researchers to farms, data regarding management factors and husbandry in studies using sent-in diagnostic samples are often less complete. Furthermore, results of all these types of studies rely on the diagnostic tools that are used to examine the samples (Boelow et al. 2022). Herein, samples were partially derived from diagnostic samples sent to the Institute for Parasitology and Tropical Veterinary Medicine (*n* = 426), partially sent in directly for the purpose of method comparison study (*n* = 469) and partially from an ongoing cross-sectional study (*n* = 172). This heterogenous origin of the samples might well be able to introduce any kind of unpredictable bias. However, the large number of samples suggests that despite some bias meaningful epidemiological information can be obtained. For strongyle nematodes and *Parascaris* spp., the two most prevalent parasite groups, the influence of potential epidemiological risk factors but also of some factors related to faecal sampling and sample handling were analysed.

For strongyle nematode egg shedding, a prevalence of 46% was observed in the study population. This is similar to what others have observed in Germany. Raue et al. (2017) have reported that between 2003 and 2012 on average 30.1% of the equine samples sent to the diagnostic service of the Institute for Parasitology at the University of Veterinary Medicine Hannover were positive. Hinney et al. (2011) performed a cross-sectional study in Brandenburg, from where slightly more than two-thirds of the samples in the present study came and found strongyle eggs in 67% of the samples collected in 2006. Important to note is that in this study samples were collected from August to early December, i.e. mainly in autumn, which also in the present study was the season with highest strongyle prevalence of over 70%. In another German-wide cross-sectional study performed in 2012/2013, 44.6% of the horse samples were positive (Schneider et al. 2014). Wirtherle et al. (2004) detected strongyle eggs in 39.8% of all samples collected in Northern Germany in 2000/2001, while von Samson-Himmelstjerna et al. (2007) observed a prevalence of strongyle egg shedding of 49.1% in North Rhine-Westphalia in 2003/2004 and Traversa et al. (2009b) investigated faecal samples from 20 German yards in 2008 with 62.8% of the samples being positive. Considering the fact that some of these studies used sedimentation/flotation while others applied various modified McMaster techniques with different multiplication factors (= theoretical limit of detection as defined by Nielsen (2021) and that different flotation solutions such as saturated ZnSO_4_ (relative density 1.3) and saturated NaCl_2_ (relative density 1.2) were used, substantial variation due to technical differences can be expected. Even with the same technique, such data can be highly variable over the years as exemplified by the data from the diagnostics in Hannover, where the prevalence of strongyle eggs ranged between 14.3% and 38.6% (Raue et al. 2017).

In the multivariate analyses conducted to identify risk factors and protective factors for the prevalence of strongyle egg shedding, the use of moxidectin for the last treatment was highly significant from the reference (pyrantel). Resistance to benzimidazoles and pyrantel is known to be widespread in strongyle nematodes of horses (Raza et al. 2019; Dauparaitė et al. 2021; Matthews 2014; Rendle et al. 2021; Coles et al. 2006; von Samson-Himmelstjerna 2012; Kenealy 2019; Stratford et al. 2011; Tzelos and Matthews 2016; Tzelos et al. 2019; Kaplan and Vidyashankar 2012) and has also been reported from Germany (Traversa et al. 2009b; Wirtherle et al. 2004). In comparison, there are only a few reports of resistance to ivermectin in strongyles (Traversa et al. 2009a; Relf et al. 2014; Geurden et al. 2013; Kaplan et al. 2004; Johnson and Biddle 2021) and moxidectin (Abbas et al. 2021; Tzelos et al. 2017; Nielsen et al. 2020; Johnson and Biddle 2021), none of them from Germany. More often than clear resistance, longer egg reappearance periods have been reported for many regions of the world (Daniels and Proudman 2016; Tzelos et al. 2017; Johnson and Biddle 2021) including Germany (von Samson-Himmelstjerna et al. 2007); and this is considered to be a hint of a developing anthelmintic resistance. However, in Brandenburg, from where the majority of samples in this study came, no hints for resistance or a prolonged egg reappearance period were observed in 2007 (Fischer et al. 2015). The fact that moxidectin treatment is associated with the longest egg reappearance period among all licenced equine anthelmintics (Relf et al. 2014; Traversa et al. 2009b; Schumacher and Taintor 2008; Johnson and Biddle 2021) and that an increased egg reappearance period (Relf et al. 2014; Traversa et al. 2009b; Schumacher and Taintor 2008; Johnson and Biddle 2021) or even resistance (Abbas et al. 2021) were only rarely reported for moxidectin is the most likely explanation for the stronger or longer lasting protective effect of moxidectin in comparison to other anthelmintics in the present study. In contrast to moxidectin, ivermectin and fenbendazole treatments were only associated with significantly lower strongyle egg-shedding data than pyrantel in the bivariate analysis. The fact that fenbendazole also had a protective effect on the odds to be positive for strongyle egg shedding at least in comparison to pyrantel in the bivariate analysis was rather surprising since resistance against benzimidazoles is well known to be a frequent problem at least on West German horse farms (Traversa et al. 2009b; Wirtherle et al. 2004). However, among the 51 samples from horses with fenbendazole as last treatment for which the age was known 25 (48.1%) were from foals, which is significantly higher than the 18.5% (139/753) for the remaining drugs (*p* < 0.0001, mid-*p* exact test). None of the samples from horses treated with fenbendazole was derived from a yearling. Foals in general had a lower prevalence while prevalence in yearlings was the highest of the three age groups. The significant effect of the last treatment with fenbendazole might therefore be an artefact caused by the fact that fenbendazole was only used for foals and adults in the analysed data set. Indeed, fenbendazole is nowadays used particularly for the deworming of foals since it is recommended for the treatment of *Strongyloides* (Reinemeyer 2009; Lind and Christensson 2009) and benzimidazole resistance is very rare in *Parascaris* spp. (Reinemeyer 2009; Lind and Christensson 2009; Peregrine et al. 2014). In contrast to the odds to be positive for strongyle egg shedding, there was no effect of the drug used for the last treatment on egg-shedding intensity.

When looking at the age groups of the horses using multivariate analyses, yearlings were significantly more often positive than adults and also had significantly higher EPGs. The comparison between adults and foals revealed only significant differences between adults and foals. Moreover, the presence of foals on the farm significantly increased the overall FECs. It is well known that horses require several years to develop full immunity against infections with strongyle nematodes leading to lower FECs (von Samson-Himmelstjerna 2012; Reinemeyer and Nielsen 2017; Hinney et al. 2011). The fact that foals did not have a significantly higher prevalence than adults can, of course, not be explained by immunological protection but may be associated with the fact that foals, while still suckling during the first months on pasture, are only gradually taking up strongyle infections during their first grazing season. Furthermore, foals in Germany are typically treated very frequently (Becher et al. 2018; von Samson-Himmelstjerna et al. 2009; Fritzen et al. 2010)—not only to reduce the burden of strongyle nematodes but in particular to prevent losses due to the highly pathogenic foal parasites *Parascaris* spp. Since migrating larvae of these parasites can lead to considerable pathology already during the prepatency period, e.g. when penetrating the alveoli in the lungs, treatment frequencies in foals can be as high as 6–12/year and were reported to be on average 4.52/year (Fritzen et al. 2010; von Samson-Himmelstjerna et al. 2007). Such high treatment frequencies are expected to also reduce any prevalence of strongyle nematodes even though most of the time fenbendazole is used. Despite the widespread occurrence of benzimidazole resistance in strongyles, the efficacy is usually not zero but only reduced. For instance, Wirtherle et al. (2004) found resistance to fenbendazole on 10/10 farms from Northwest Germany, but the observed FECR was between 55 and 94% indicating that anthelmintic treatment usually still exerts some effects on farms with resistance.

The presence of foals on a farm significantly increased strongyle prevalence as well as egg-shedding intensity in the bivariate analyses. In contrast, the number of foals had a significant but small negative effect only on egg-shedding intensity. In the multivariate analyses, a small but significant positive effect of the number of foals on strongyle egg-shedding prevalence and a positive effect of the presence of foals on strongyle egg-shedding intensity were calculated. Obviously, the variable presence of foals and the number of foals are highly correlated, which might explain the complex pattern of results in bi- and multivariate analyses. In any case, foals on a farm overall led to a higher risk that strongyle eggs would be shed. Since the prevalence and egg-shedding intensity of foals themselves were not very high, it is likely that effects were indirect, e.g. by differences in management between stud farms and riding stables, high numbers of yearlings on farms that also had foals (Kuzmina et al. 2016; Nielsen et al. 2018).

In the bivariate analyses, clear differences between the seasons were found for both strongyle egg-shedding prevalence and intensity data. In both analyses, spring was the season with the lowest odds/risk of strongyle egg shedding while the highest prevalence and intensity were observed in autumn. In the multivariate analyses, this pattern was confirmed. In recent years, there have been two studies from Great Britain on the seasonality of strongyle egg shedding, one of which found an increased occurrence of strongyle eggs in the summer and a reduction in the winter months (Wood et al. 2013). A Swedish study confirmed the increased egg excretion in summer (Tydén et al. 2019). The other British study detected no seasonality of strongyle egg shedding (Lester et al. 2018; Steuer et al. 2022). A recent study by Nielsen et al. (2022) recommends that strongyles are best examined in spring or winter.

The variable sex had no significant effect in the analyses on the presence/absence of strongyle egg shedding nor in the bivariate analyses on egg-shedding intensity or abundance. In contrast, it turned out to have a significant influence on egg-shedding intensity in the multivariate analysis with females showing significantly higher strongyle EPGs than males. However, data about sex in the data set must be considered with a lot of care since horse owners obviously did not provide reliable information regarding the number of male horses that were neutered. Thus, the category “male” includes intact male foals and young yearlings before sexual maturity, presumably a few sexually mature stallions and a large number of neutered geldings. Without precise information about the status of the individual horses in the study population, any analysis of the effects of sex on nematode egg shedding cannot be considered to be reliable.

Similarly, the state of origin is a problematic variable since one state (Brandenburg) is highly overrepresented (64.9% of all samples) while four out of nine states are represented by six or less samples. Since the glm() function creates dummy variables for all the states, the inclusion of the variable “state” leads to a considerable decrease in the precision with which all other variables in the model can be estimated. Thus, although there are obviously significant differences in the bivariate analyses, the data set is simply ill-suited to analyse geographic differences and a sampling technique that results in representative sampling for each state included would be required.

Regarding the variable “number of horses”, which had a very weak negative influence on the egg-shedding intensity only in the multivariate analysis, it is difficult to find a convincing explanation. Intuitively, one would think that high numbers of horses might increase the infection pressure and thus lead to a higher prevalence and abundance of parasites. This consideration is obvious for natural populations of hosts such as wildlife (Slivinska et al. 2020; Harvey et al. 2019) but does not necessarily describe the situation in managed populations of domestic animals. One simple explanation why there was a tendency to find the opposite is that farms with larger numbers of animals have an overall better/more professional management including hygiene measures. However, published data do not support the latter explanation (von Samson-Himmelstjerna et al. 2009; Fritzen et al. 2010).

Finally, two technical aspects were shown to be relevant for the results. The way samples were collected had a significant impact on the odds to be positive and also on the observed strongyle egg-shedding intensity. In order to improve sensitivity to detect parasites showing unregular egg shedding, such as liver flukes but also some parasitic nematodes, it is often recommended to collect samples over several (typically three to five) consecutive days and perform the diagnosis on the pooled sample (Nielsen et al. 2021; Bracken et al. 2012; Bredtmann et al. 2017b). Surprisingly, however, composite samples showed significantly lower prevalence and egg-shedding intensity in both the bi- and multivariate analyses. This suggests that composite samples decrease the sensitivity of strongyle egg detection instead of increasing it. One simple explanation could be related to the storage of samples during the collection days. If the samples are not cooled during storage as recommended, it is expected that, depending on the environmental temperatures, a substantial fraction of the eggs develops into larvae that hatch and are only inefficiently floated (Nielsen et al. 2010c; Jagła et al. 2013). This is not expected to be relevant for eggs of trematodes, cestodes and ascarides that do not hatch spontaneously in faecal samples but might well be a problem for strongyles. In order to confirm such a negative impact of sampling over multiple days on the sensitivity to diagnose strongyle eggs, paired single and composite subsamples from the same faecal sample should be compared to obtain direct evidence and improve recommendations for sampling strategies.

The second variable related to technical aspects is the shipping time. Prolonged shipping time is also expected to have a negative effect on strongyle egg prevalence and counts since larvae might hatch during transport at ambient temperature. Indeed, bivariate analyses confirmed this expectation since shipping time had significant negative effects on the odds of samples to be positive for strongyle eggs and on the FECs. However, in the final logistic regression model, the shipping time was not included, and in the negative binomial regression model, the negative effect was not significant. This is most likely explainable by the fact that the vast majority of the samples had short shipping times and the power of the analyses to detect effects of shipping time on egg-shedding prevalence and intensity in a multivariate model was too low.

The analyses of the data for *Parascaris* spp. egg-shedding prevalence and intensity suffer from the problem that the overall number of positive samples was rather small and this led to large confidence intervals for odds and risk ratios of some influencing factors and a limited number of significant comparisons.

*Parascaris* spp. are well known to be predominantly parasites of foals and to some extent also of yearlings (Fritzen et al. 2010; Southwood et al. 1998; Rehbein et al. 2013; von Samson-Himmelstjerna et al. 2007; Hautala et al. 2019; Clayton and Duncan 1979a; von Samson-Himmelstjerna 2012). Thus, it was not surprising that several of the variables with influence on *Parascaris* spp. prevalence and egg-shedding intensity were related to the presence of foals. The facts that *Parascaris* spp. eggs were exclusively detected in samples originating from farms with foals and that prevalence observed in foals was approximately 5.4- and 61-fold higher than in yearlings and adults clearly demonstrate the high relevance of this parasite for very young horses. The analysis of data for *Parascaris* spp. clearly revealed that foals had much higher odds to be positive for *Parascaris* spp. eggs in both bivariate and multivariate analyses. Regarding FECs, there was also a higher abundance in foals than in the other age groups, but differences in intensity were not significant. Again, the variables “presence of foals” and “number of foals” are highly correlated in the multivariate analysis, and due to the small number of *Parascaris* spp. positive samples, this might lead to unexpected results of the statistical analyses. While the number of foals actually had a very small but significant negative effect on the odds to be *Parascaris* spp. positive in the multi-variate analysis, the odds ratio for “presence of foals” was very high but the 95% CI was also very wide. A completely different pattern was observed for egg-shedding intensity, with both variables “presence of foals” and “number of foals” having no effect in the multi-variate analysis.

In the bi- and multivariate analyses, there were also significant effects of the time since the last treatment of the horse. Surprisingly, it was protective if the last treatment was more than 8 weeks ago. However, this effect was only observed for prevalence data in the bivariate and for intensity data in the multi-variate analysis. This unexpected protective effect is presumably caused by the fact that almost all *Parascaris* spp. positive animals were foals and that foals had much higher treatment frequencies than yearlings and adults. Effects of different anthelmintics were only significant in the bivariate but not the multivariate analysis regarding the prevalence of *Parascaris* spp. Presumably, this is due to the small number of positive horses and the large number of six different drugs or drug combinations that were used. In the bivariate analysis, prevalence was highest for fenbendazole followed by pyrantel, moxidectin and ivermectin. No significant effects were found on egg-shedding intensity. Since resistance to ivermectin is well known to be widespread at least in Western Germany (von Samson-Himmelstjerna et al. 2007) and Central Europe (Traversa et al. 2009a; Relf et al. 2014; Geurden et al. 2013; Johnson and Biddle 2021), the finding that ivermection provides better protection than any of the other drugs is somewhat surprising, and on the farms with foals in the study population ivermectin resistance was apparently no major problem. Moreover, macrocyclic lactones are able to eliminate tissue migrating larval stages of *Parascaris* spp. (Reinemeyer and Nielsen 2017; Lyons and Tolliver 2012; Lindgren et al. 2008) and this property is expected to cause the better/longer lasting protection than pyrantel and fenbendazole, which are both only active against gut luminal stages of the parasite (Luksovsky et al. 2013; Armstrong et al. 2014; Lindgren et al. 2008).

The variables “sex” and “state of origin” also showed significant effects on the egg-shedding intensity in the multivariate model and the prevalence of *Parascaris* spp. egg shedding in the bivariate analysis, respectively. However, due to the reasons already explained above for strongyle data, the current data set is not suitable to analyse the effect of these variables.

In conclusion, the analysis of the present data set provides important information about prevalence and egg-shedding intensity of strongyle and ascarid nematodes in the German horse population. The identified risk factors varied considerably depending on whether data for strongyles or *Parascaris* spp. were analysed but also between prevalence and egg-shedding intensity. However, the season when samples were collected, the age group as well as the presence of foals on the yard were among the variables that often influenced the data. The drug used for the last treatment was only relevant for prevalence but not for egg-shedding intensity.

## Supplementary Information

Below is the link to the electronic supplementary material.

Supplementary file1 (PDF 117 KB)

Supplementary file2 (DOCX 13 KB)

Supplementary file3 (PDF 115 KB)

Supplementary file4 (PDF 112 KB)

Supplementary file5 (PDF 86 KB)

Supplementary file6 (PDF 147 KB)

Supplementary file7 (PDF 147 KB)

Supplementary file8 (PDF 82 KB)

## Data Availability

All data generated or analysed during this study are included in this article and in the manuskript “Comparison of FECPAK^G2^, a modified Mini-FLOTAC technique and combined sedimentation and flotation for the coproscopic examination of helminth eggs in horses”. https://doi.org/10.1186/s13071-022-05266-y

## Abbreviations

EPG: Egg per gramme faeces
FEC: Faecal egg count
AIC: Akaike information criterion

## References

[CR1] Abbas G, Ghafar A, Hurley J, Bauquier J, Beasley A, Wilkes EJA, Jacobson C, El-Hage C, Cudmore L, Carrigan P, Tennent-Brown B, Gauci CG, Nielsen MK, Hughes KJ, Beveridge I, Jabbar A (2021). Cyathostomin resistance to moxidectin and combinations of anthelmintics in Australian horses. Parasit Vectors.

[CR2] Armstrong SK, Woodgate RG, Gough S, Heller J, Sangster NC, Hughes KJ (2014). The efficacy of ivermectin, pyrantel and fenbendazole against Parascaris equorum infection in foals on farms in Australia. Vet Parasitol.

[CR3] Becher AM, Van Doorn DC, Pfister K, Kaplan RM, Reist M, Nielsen MK (2018). Equine parasite control and the role of national legislation - a multinational questionnaire survey. Vet Parasitol.

[CR4] Bellaw JL, Nielsen MK (2020). Meta-analysis of cyathostomin species-specific prevalence and relative abundance in domestic horses from 1975–2020: emphasis on geographical region and specimen collection method. Parasit Vectors.

[CR5] Boelow H, Krücken J, Thomas E, Mirams G, Von Samson-Himmelstjerna G (2022). Comparison of FECPAK^G2^, a modified Mini-FLOTAC technique and combined sedimentation and flotation for the coproscopic examination of helminth eggs in horses. Parasit Vectors.

[CR6] Bracken MK, Wøhlk CBM, Petersen SL, Nielsen MK (2012). Evaluation of conventional PCR for detection of Strongylus vulgaris on horse farms. Vet Parasitol.

[CR7] Bredtmann CM, Krücken J, Murugaiyan J, Kuzmina T, Von Samson-Himmelstjerna G (2017). Nematode species identification-current status, challenges and future perspectives for cyathostomins. Front Cell Infect Microbiol.

[CR8] Bredtmann CM, Krücken J, Murugaiyan J, Kuzmina T & Von Samson-Himmelstjerna G (2017b) Nematode species identification—current status, challenges and future perspectives for cyathostomins. Frontiers in Cellular and Infection Microbiology, 7 10.3389/fcimb.2017.00283 PMC5487379 28702376

[CR9] Bucknell DG, Gasser RB, Beveridge I (1995). The prevalence and epidemiology of gastrointestinal parasites of horses in Victoria, Australia. Int J Parasitol.

[CR10] Clayton HM (1986). Ascarids. Recent advances. Vet Clin North Am Equine Pract.

[CR11] Clayton HM, Duncan JL (1979). The development of immunity to *Parascaris equorum* infection in the foal. Res Vet Sci.

[CR12] Clayton HM, Duncan JL (1979). The migration and development of *Parascaris equorum* in the horse. Int J Parasitol.

[CR13] Coles GC, Jackson F, Pomroy WE, Prichard RK, Von Samson-Himmelstjerna G, Silvestre A, Taylor MA, Vercruysse J (2006). The detection of anthelmintic resistance in nematodes of veterinary importance. Vet Parasitol.

[CR14] Corning S (2009). Equine cyathostomins: a review of biology, clinical significance and therapy. Parasit Vectors.

[CR15] Daniels SP, Proudman CJ (2016). Shortened egg reappearance after ivermectin or moxidectin use in horses in the UK. Vet J.

[CR16] Dauparaitė E, Kupčinskas T, Von Samson-Himmelstjerna G, Petkevičius S (2021). Anthelmintic resistance of horse strongyle nematodes to ivermectin and pyrantel in Lithuania. Acta Vet Scand.

[CR17] Ertelt A, Merle R, Samson-Himmelstjerna GV, Wulke N, Demeler J, Gehlen H (2015). Management factors and their impact on helminthic fecal egg count in horses. Pferdeheilkunde Equine Medicine.

[CR18] Fischer JK, Hinney B, Denwood MJ, Traversa D, Von Samson-Himmelstjerna G, Clausen PH (2015). Efficacy of selected anthelmintic drugs against cyathostomins in horses in the federal state of Brandenburg, Germany. Parasitol Res.

[CR19] Fritzen B, Rohn K, Schneider T, Von Samson-Himmelstjerna G (2010). Endoparasite control management on horse farms – lessons from worm prevalence and questionnaire data. Equine Vet J.

[CR20] Geurden T, Betsch JM, Maillard K, Vanimisetti B, D'espois M, Besognet B (2013). Determination of anthelmintic efficacy against equine cyathostomins and *Parascaris equorum* in France. Equine Vet Educ.

[CR21] Harvey AM, Meggiolaro MN, Hall E, Watts ET, Ramp D, Šlapeta J (2019). Wild horse populations in south-east Australia have a high prevalence of Strongylus vulgaris and may act as a reservoir of infection for domestic horses. Int J Parasitol Parasites Wildl.

[CR22] Hautala K, Näreaho A, Kauppinen O, Nielsen MK, Sukura A, Rajala-Schultz PJ (2019). Risk factors for equine intestinal parasite infections and reduced efficacy of pyrantel embonate against *Parascaris* sp. Vet Parasitol.

[CR23] Hinney B, Wirtherle NC, Kyule M, Miethe N, Zessin K-H, Clausen P-H (2011). Prevalence of helminths in horses in the state of Brandenburg Germany. Parasitol Res.

[CR24] Jagła E, Śpiewak J, Zaleśny G, Popiołek M (2013). Effect of storage and preservation of horse faecal samples on the detectability and viability of strongylid nematode eggs and larvae. Bull Vet Inst Pulawy.

[CR25] Johnson ACB & Biddle AS (2021) The use of molecular profiling to track equine reinfection rates of cyathostomin species following anthelmintic administration. 538kürzere Zeiträume für wiederauftreten von Eiern, 11 10.3390/ani11051345 PMC8150961 34065099

[CR26] Jürgenschellert L, Krücken J, Austin CJ, Lightbody KL, Bousquet E & Von Samson-Himmelstjerna G (2020) Investigations on the occurrence of tapeworm infections in German horse populations with comparison of different antibody detection methods based on saliva and serum samples. Parasit Vectors, 13 10.1186/s13071-020-04318-5 PMC7488081 32912340

[CR27] Kaplan RM (2002). Anthelmintic resistance in nematodes of horses. Vet Res.

[CR28] Kaplan RM, Vidyashankar AN (2012). An inconvenient truth: global worming and anthelmintic resistance. Vet Parasitol.

[CR29] Kaplan RM, Klei TR, Lyons ET, Lester G, Courtney CH, French DD, Tolliver SC, Vidyashankar AN, Zhao Y (2004). Prevalence of anthelmintic resistant cyathostomes on horse farms. J Am Vet Med Assoc.

[CR30] Kenealy JS (2019) Anthelmintic resistance in equine parasites: mechanism and treatment approaches. 2019

[CR31] Kuzmina TA, Dzeverin I, Kharchenko VA (2016). Strongylids in domestic horses: influence of horse age, breed and deworming programs on the strongyle parasite community. Vet Parasitol.

[CR32] Lester HE, Morgan ER, Hodgkinson JE, Matthews JB (2018). Analysis of strongyle egg shedding consistency in horses and factors that affect it. J Equine Vet.

[CR33] Lind EO, Christensson D (2009). Anthelmintic efficacy on Parascaris equorum in foals on Swedish studs. Acta Vet Scand.

[CR34] Lindgren K, Ljungvall Ö, Nilsson O, Ljungström BL, Lindahl C, Höglund J (2008). Parascaris equorum in foals and in their environment on a Swedish stud farm, with notes on treatment failure of ivermectin. Vet Parasitol.

[CR35] Luksovsky J, Craig TM, Bingham GM, Cyr T, Forrest D (2013). Determining treatment to control two multidrug-resistant parasites on a Texas horse farm. J Equine Vet.

[CR36] Lyons ET, Tolliver SC (2012). Macrocyclic lactones for parasite control in equids. Curr Pharm Biotechnol.

[CR37] Lyons ET, Tolliver SC, Kuzmina TA, Collins SS (2011). Further evaluation in field tests of the activity of three anthelmintics (fenbendazole, oxibendazole, and pyrantel pamoate) against the ascarid *Parascaris equorum* in horse foals on eight farms in Central Kentucky (2009–2010). Parasitol Res.

[CR38] Matthee S (2003). Anthelmintic treatment in horses: the extra-label use of products and the danger of under-dosing. J S Afr Vet Assoc.

[CR39] Matthews JB (2014). Anthelmintic resistance in equine nematodes. Int J Parasitol Drugs Drug Resist.

[CR40] Nielsen MK (2016). Evidence-based considerations for control of *Parascaris* spp. infections in horses. Equine Vet Educ.

[CR41] Nielsen MK (2021). What makes a good fecal egg count technique?. Vet Parasitol.

[CR42] Nielsen MK, Baptiste KE, Tolliver SC, Collins SS, Lyons ET (2010). Analysis of multiyear studies in horses in Kentucky to ascertain whether counts of eggs and larvae per gram of feces are reliable indicators of numbers of strongyles and ascarids present. Vet Parasitol.

[CR43] Nielsen MK, Fritzen B, Duncan JL, Guillot J, Eysker M, Dorchies P, Laugier C, Beugnet F, Meana A, Lussot-Kervern I, Von Samson-Himmelstjerna G (2010). Practical aspects of equine parasite control: a review based upon a workshop discussion consensus. Equine Vet J.

[CR44] Nielsen MK, Vidyashankar AN, Andersen UV, Delisi K, Pilegaard K, Kaplan RM (2010). Effects of fecal collection and storage factors on strongylid egg counts in horses. Vet Parasitol.

[CR45] Nielsen MK, Pfister K, Von Samson-Himmelstjerna G (2014). Selective therapy in equine parasite control–application and limitations. Vet Parasitol.

[CR46] Nielsen MK, Reinemeyer CR, Donecker JM, Leathwick DM, Marchiondo AA, Kaplan RM (2014). Anthelmintic resistance in equine parasites—current evidence and knowledge gaps. Vet Parasitol.

[CR47] Nielsen MK, Branan MA, Wiedenheft AM, Digianantonio R, Scare JA, Bellaw JL, Garber LP, Kopral CA, Phillippi-Taylor AM, Traub-Dargatz JL (2018). Risk factors associated with strongylid egg count prevalence and abundance in the United States equine population. Vet Parasitol.

[CR48] Nielsen MK, Banahan M, Kaplan RM (2020). Importation of macrocyclic lactone resistant cyathostomins on a US thoroughbred farm. Int J Parasitol Drugs Drug Resist.

[CR49] Nielsen MK, Facison C, Scare JA, Martin AN, Gravatte HS, Steuer AE (2021). Diagnosing Strongylus vulgaris in pooled fecal samples. Vet Parasitol.

[CR50] Nielsen MK, Mittel L, Grice A, Erskine M, Graves E, Vaala W, Tully RC, Drench DD, Bowman R & M. K (2019). AAEP Internal Parasite Control Guidelines. In: Practitioners, American association of equine (ed.)

[CR51] Nielsen MK, Von Samson-Himmelstjerna G, Kuzmina TA, Van Doorn DCK, Meana A, Rehbein S, Elliott T & Reinemeyer CR (2022) World association for the advancement of veterinary parasitology (WAAVP): Third edition of guideline for evaluating the efficacy of equine anthelmintics. Vet Parasitol 303:109676 10.1016/j.vetpar.2022.109676 35164972

[CR52] Peregrine AS, Molento MB, Kaplan RM, Nielsen MK (2014). Anthelmintic resistance in important parasites of horses: does it really matter?. Vet Parasitol.

[CR53] Raue K, Heuer L, Böhm C, Wolken S, Epe C, Strube C (2017). 10-year parasitological examination results (2003 to 2012) of faecal samples from horses, ruminants, pigs, dogs, cats, rabbits and hedgehogs. Parasitol Res.

[CR54] Raza A, Qamar AG, Hayat K, Ashraf S, Williams AR (2019). Anthelmintic resistance and novel control options in equine gastrointestinal nematodes. Parasitology.

[CR55] Rehbein S, Visser M, Winter R (2013). Prevalence, intensity and seasonality of gastrointestinal parasites in abattoir horses in Germany. Parasitol Res.

[CR56] Reinemeyer CR (2009). Diagnosis and control of anthelmintic-resistant *Parascaris equorum*. Parasit Vectors.

[CR57] Reinemeyer CR (2012). Anthelmintic resistance in non-strongylid parasites of horses. Vet Parasitol.

[CR58] Reinemeyer CR, Nielsen MK (2009). Parasitism and colic. Vet Clin North Am Equine Pract.

[CR59] Reinemeyer CR, Nielsen MK (2017). Control of helminth parasites in juvenile horses. Equine Veterinary Education.

[CR60] Relf VE, Morgan ER, Hodgkinson JE, Matthews JB (2013). Helminth egg excretion with regard to age, gender and management practices on UK Thoroughbred studs. Parasitology.

[CR61] Relf VE, Lester HE, Morgan ER, Hodgkinson JE, Matthews JB (2014). Anthelmintic efficacy on UK Thoroughbred stud farms. Int J Parasitol.

[CR62] Rendle D, Mountford D, Roberts C, Owers R, Mair T, Bowen M, Matthews J, Richards I, Hodgkinson J, Furtado T, Sharpe L, Frost R (2021). Anthelmintic resistance in equids. Vet Rec.

[CR63] Saeed MA, Beveridge I, Abbas G, Beasley A, Bauquier J, Wilkes E, Jacobson C, Hughes KJ, El-Hage C, O’handley R, Hurley J, Cudmore L, Carrigan P, Walter L, Tennent-Brown B, Nielsen MK & Jabbar A,  (2019). Systematic review of gastrointestinal nematodes of horses from Australia. Parasit Vectors.

[CR64] Scheuerle MC, Stear MJ, Honeder A, Becher AM, Pfister K (2016). Repeatability of strongyle egg counts in naturally infected horses. Vet Parasitol.

[CR65] Schneider S, Pfister K, Becher AM, Scheuerle MC (2014). Strongyle infections and parasitic control strategies in German horses - a risk assessment. BMC Vet Res.

[CR66] Schumacher J, Taintor J (2008). A review of the use of moxidectin in horses. Equine Vet Educ.

[CR67] Slivinska K, Klich D, Yasynetska N, Żygowska M (2020). The effects of seasonality and group size on fecal egg counts in wild Przewalski’s Horses (Equus Ferus Przewalskii, Poljakov, 1881) in The Chernobyl Exclusion Zone, Ukraine During 2014–2018. Helminthologia.

[CR68] Southwood LL, Baxter GM, Bennett DG & Ragle CA (1998) Ascarid impaction in young horses. Compendium on continuing education for the practicing veterinarian 20:100-+

[CR69] Steuer AE, Anderson HP, Shepherd T, Clark M, Scare JA, Gravatte HS, Nielsen MK (2022). Parasite dynamics in untreated horses through one calendar year. Parasit Vectors.

[CR70] Stratford CH, Mcgorum BC, Pickles KJ & Matthews JB (2011) An update on cyathostomins: anthelmintic resistance and diagnostic tools. Equine Vet J Suppl 133–139 10.1111/j.2042-3306.2011.00397.x 21790768

[CR71] Traversa D, Iorio R, Otranto D, Giangaspero A, Milillo P, Klei TRL (2009). Species-specific identification of equine cyathostomes resistant to fenbendazole and susceptible to oxibendazole and moxidectin by macroarray probing. Exp Parasitol.

[CR72] Traversa D, Von Samson-Himmelstjerna G, Demeler J, Milillo P, Schürmann S, Barnes H, Otranto D, Perrucci S, Di Regalbono AF, Beraldo P, Boeckh A, Cobb R (2009). Anthelmintic resistance in cyathostomin populations from horse yards in Italy United Kingdom and Germany. Parasit Vectors.

[CR73] Tydén E, Jansson A & Ringmark S (2019) Parasites in Horses Kept in A 2.5 Year-Round Grazing System in Nordic Conditions without Supplementary Feeding. Animals : an open access journal from MDPI, 9:1156 10.3390/ani9121156 PMC6940839 31861066

[CR74] Tzelos T, Matthews J (2016). Anthelmintic resistance in equine helminths and mitigating its effects. In Pract.

[CR75] Tzelos T, Barbeito JSG, Nielsen MK, Morgan ER, Hodgkinson JE, Matthews JB (2017). Strongyle egg reappearance period after moxidectin treatment and its relationship with management factors in UK equine populations. Vet Parasitol.

[CR76] Tzelos T, Morgan ER, Easton S, Hodgkinson JE, Matthews JB (2019). A survey of the level of horse owner uptake of evidence-based anthelmintic treatment protocols for equine helminth control in the UK. Vet Parasitol.

[CR77] Von Samson-Himmelstjerna G (2012). Anthelmintic resistance in equine parasites - detection, potential clinical relevance and implications for control. Vet Parasitol.

[CR78] Von Samson-Himmelstjerna G, Fritzen B, Demeler J, Schürmann S, Rohn K, Schnieder T, Epe C (2007). Cases of reduced cyathostomin egg-reappearance period and failure of *Parascaris equorum* egg count reduction following ivermectin treatment as well as survey on pyrantel efficacy on German horse farms. Vet Parasitol.

[CR79] Von Samson-Himmelstjerna G, Traversa D, Demeler J, Rohn K, Milillo P, Schurmann S, Lia R, Perrucci S, Di Regalbono AF, Beraldo P, Barnes H, Cobb R, Boeckh A (2009). Effects of worm control practices examined by a combined faecal egg count and questionnaire survey on horse farms in Germany Italy and the UK. Parasit Vectors.

[CR80] Wirtherle N, Schnieder T, Von Samson-Himmelstjerna G (2004). Prevalence of benzimidazole resistance on horse farms in Germany. Vet Rec.

[CR81] Wood EL, Matthews JB, Stephenson S, Slote M, Nussey DH (2013). Variation in fecal egg counts in horses managed for conservation purposes: individual egg shedding consistency, age effects and seasonal variation. Parasitology.

[CR82] Zanet S, Battisti E, Labate F, Oberto F & Ferroglio E (2021) Reduced efficacy of fenbendazole and pyrantel pamoate treatments against intestinal nematodes of stud and performance horses. Vet Sci 8 10.3390/vetsci8030042 PMC8001109 33807857

