# Supplementary material for: Epidemiological study on factors influencing the occurrence of helminth eggs in horses in Germany based on sent-in diagnostic samples
Source: Parasitol Res. 2023 Jan 11;122(3):749–67. doi: 10.1007/s00436-022-07765-4 (PMC9988789; doi:10.1007/s00436-022-07765-4)
Supplement: Supplementary file 1 — Supplementary file1 (PDF 117 KB) [file 436_2022_7765_MOESM1_ESM.pdf]

Supplementary Table S1 Effect of continuous variables on prevalence of strongyle egg shedding in bivariate logistic regression models

Variable	Estimate[c]	SE[a]	OR[b] (95% CI)	p value
Number of horses	-0.0011	0.0003	0.9989 (0.9982-0.9996)	0.0017
Number of foals	0.0021	0.0015	1.0021 (0.9992-1.0050)	0.152
Shipping time	-0.0100	0.0098	0.9900 (0.9712-1.0092)	0.306

[a]SE, standard error
[b]Odds ratio with 95% confidence interval
[c]Result of t test for differences in estimates
