# Supplementary material for: Epidemiological study on factors influencing the occurrence of helminth eggs in horses in Germany based on sent-in diagnostic samples
Source: Parasitol Res. 2023 Jan 11;122(3):749–67. doi: 10.1007/s00436-022-07765-4 (PMC9988789; doi:10.1007/s00436-022-07765-4)
Supplement: Supplementary file 2 — Supplementary file2 (DOCX 13 KB) [file 436_2022_7765_MOESM2_ESM.docx]

**Supplementary Table S2** **Effect of continuous variables on abundance and intensity of strongyle egg shedding in bivariate logistic regression models analysed by Spearman correlation**

|  | Abundance | | Intensity | |
| --- | --- | --- | --- | --- |
| Variable | Rho | p value | Rho | p value |
| Number of horses | -0.078 | 0.016 | -0.191 | 0.708 |
| Number of foals | 0.091 | 0.013 | 0.092 | 0.102 |
| Shipping time (days) | -0.109 | <0.001 | -0.110 | 0.022 |
