# Supplementary material for: Epidemiological study on factors influencing the occurrence of helminth eggs in horses in Germany based on sent-in diagnostic samples
Source: Parasitol Res. 2023 Jan 11;122(3):749–67. doi: 10.1007/s00436-022-07765-4 (PMC9988789; doi:10.1007/s00436-022-07765-4)
Supplement: Supplementary file 3 — Supplementary file3 (PDF 115 KB) [file 436_2022_7765_MOESM3_ESM.pdf]

Supplementary Table S3 Logistic regression model describing risk factors associated with positive diagnosis of strongyle eggs by sedimentation/flotation

Variable	Level	Estimate	SE[a]	OR[b]	95% CI[c]	p value[d]
Season	Spring	Ref.			1	
	Summer	1.00	0.27	2.71	1.60-4.66	0.00025
	Autumn	2.24	0.35	9.41	4.85-18.87	9.23^{E-11}
	Winter	1.28	0.26	3.61	2.16-6.11	1.19^{E-6}
Last treatment	Pyrantel	Ref.			1	
	Ivermectin	-0.33	0.28	0.72	0.41-1.25	0.244
	Moxidectin	-2.10	0.39	0.12	0.06-0.26	6.19^{E-8}
	Doramectin	13.93	624.47	1116171.91	0.00-n.a.	0.982
	Fenbendazole	-0.57	0.45	0.56	0.23-1.35	0.204
	Ivermectin/Pyrantel	-0.13	0.73	0.88	0.23-4.34	0.858
Age group	Foals (<1 year)	-0.22	0.28	0.80	0.46-1.39	0.436
	Yearlings (1-4 years)	1.38	0.36	3.98	2.02-8.23	0.0001
	Adults (>4 years)	Ref.			1	
Foals no.		0.04	0.01	1.04	1.01-1.06	0.006
Sample type	Individual	Ref.			1	
	Composite	-1.00	0.51	0.37	0.13-0.98	0.0499

Number of observations in the model: 592
AIC = 671.99, Nagelkerke's R^2 = 0.339. Tjur's R^2 = 0.262
[a]SE, standard error.
[b]OR, odds ratio.
[c]CI, confidence interval.
[d]Result of t test.
Ref., reference level.
