# Supplementary material for: Epidemiological study on factors influencing the occurrence of helminth eggs in horses in Germany based on sent-in diagnostic samples
Source: Parasitol Res. 2023 Jan 11;122(3):749–67. doi: 10.1007/s00436-022-07765-4 (PMC9988789; doi:10.1007/s00436-022-07765-4)
Supplement: Supplementary file 4 — Supplementary file4 (PDF 112 KB) [file 436_2022_7765_MOESM4_ESM.pdf]

Supplementary Table S4 Zero-truncated negative binomial regression model describing risk factors associated with intensity of strongyle egg shedding as determined by Mini-FLOTAC

Variable	Level	Estimate	SE[a]	RR[d]	95% CI[b]	p value[c]
Season	Spring			1		
	Summer	1.20	0.24	3.33	2.09-5.30	<0.0001
	Autumn	0.49	0.20	1.63	1.10-2.44	0.015
	Winter	0.48	0.21	1.61	1.07-2.41	0.021
Age group	Foals (<1 year)	1.03	0.20	2.81	1.91-4.12	<0.001
	Yearlings (1-4 years)	0.61	0.23	1.84	1.18-2.88	0.007
	Adults (>4 years)	Ref.		1		
Number of horses		-0.005	0.002	0.995	0.992-0.998	0.004
Sex	Male	Ref.				
	Female	0.48	0.15	1.62	1.20-2.19	0.001
Sample type	Individual	Ref.		1		
	Composite	-1.70	0.41	0.18	0.08-0.41	<0.0001

Number of observations in the model: 366
AIC = 4556.58, Nagelkerke's R^2 = 0.166
[a]SE, standard error.
[b]CI, confidence interval.
[c]Result of t test.
[d]RR, rate ratio.
Ref., reference level
