# Supplementary material for: Epidemiological study on factors influencing the occurrence of helminth eggs in horses in Germany based on sent-in diagnostic samples
Source: Parasitol Res. 2023 Jan 11;122(3):749–67. doi: 10.1007/s00436-022-07765-4 (PMC9988789; doi:10.1007/s00436-022-07765-4)
Supplement: Supplementary file 5 — Supplementary file5 (PDF 86 KB) [file 436_2022_7765_MOESM5_ESM.pdf]

Supplementary Table S5 Effect of continuous variables on intensity of *Parascaris* spp. egg shedding in bivariate logistic regression models analysed by Spearman correlation

	Abundance		Intensity	
Variable	Rho	p value	Rho	p value
Number horses	0.041	0.211	-0.076	0.631
Number foals	0.182	<0.001	-0.020	0.902
Shipping time	-0.042	0.171	0.161	0.301
