# Supplementary material for: Epidemiological study on factors influencing the occurrence of helminth eggs in horses in Germany based on sent-in diagnostic samples
Source: Parasitol Res. 2023 Jan 11;122(3):749–67. doi: 10.1007/s00436-022-07765-4 (PMC9988789; doi:10.1007/s00436-022-07765-4)
Supplement: Supplementary file 6 — Supplementary file6 (PDF 147 KB) [file 436_2022_7765_MOESM6_ESM.pdf]

Supplementary Table S6 Logistic regression model describing risk factors associated with positive diagnosis of *Parascaris* spp. eggs by sedimentation/flotation

Variable	Level	Estimate	SE[a]	OR[d]	95% CI[b]	p value[c]
Time since last treatment	< 8 weeks	Ref.		1		
	≥ 8 weeks	0.79	0.36	2.21	1.10-4.51	0.027
Age group	Foals (<1 year)	Ref.		1		
	Yearlings (1-4 years)	-2.43	0.77	0.09	0.01-0.32	0.002
	Adults (>4 years)	-3.68	0.76	0.03	0.004-0.09	<0.0001
Foals no.		-0.04	0.01	0.96	0.93-0.98	0.002
Presence of foals	No	Ref.		1		
	Yes	16.81	999.17	1.99×10^7	0-n.a.	

Number of observations in the model: 681
AIC = 235,8, Nagelkerke's R^2 = 0.379. Tjur's R^2 = 0.189.
[a]SE, standard error.
[b]CI, confidence interval.
[c]Result of t test.
[d]OR, odds ratio.
n.a., not available.
