# Supplementary material for: Epidemiological study on factors influencing the occurrence of helminth eggs in horses in Germany based on sent-in diagnostic samples
Source: Parasitol Res. 2023 Jan 11;122(3):749–67. doi: 10.1007/s00436-022-07765-4 (PMC9988789; doi:10.1007/s00436-022-07765-4)
Supplement: Supplementary file 7 — Supplementary file7 (PDF 147 KB) [file 436_2022_7765_MOESM7_ESM.pdf]

Supplementary Table S7 Negative binomial regression model describing risk factors associated with intensity of *Parascaris* spp. egg shedding as determined by Mini-FLOTAC

Variable	Level	Estimate	SE[a]	RR[d]	95% CI[b]	p value[c]
Season	Spring	Ref		1		
	Summer	2.88	0.81	17.82	3.64-87.4	<0.0001
	Autumn	2.80	0.56	16.47	5.51-49.24	<0.0001
	Winter	1.89	0.33	3.28	1.70-6.33	0.0003
Age group	Foals (<1 year)	Ref.		1		
	Yearlings (1-4 years)	-3.80	0.89	0.02	0.004-0.13	<0.0001
	Adults (>4 years)	-2.77	1.05	0.06	0.008-0.49	0.008
Sex	Male	Ref.		1		
	Female	0.62	0.35	1.87	0.95-3.68	0.071
Foals no.		-0.0009	0.02	0.99	0.96-1.02	0.555

Number of observations in the model: 566
AIC 383.34, Nagelkerke's R^2 = 0.590.
[a]SE, standard error.
[b]CI, confidence interval.
[c]Result of t test.
[d]RR, rate ratio.
n.a., not available.
