# Supplementary material for: Epidemiological study on factors influencing the occurrence of helminth eggs in horses in Germany based on sent-in diagnostic samples
Source: Parasitol Res. 2023 Jan 11;122(3):749–67. doi: 10.1007/s00436-022-07765-4 (PMC9988789; doi:10.1007/s00436-022-07765-4)
Supplement: Supplementary file 8 — Supplementary file8 (PDF 82 KB) [file 436_2022_7765_MOESM8_ESM.pdf]

Supplemental Text S1 Query form

EXAMINATION REQUEST

Sender:
Name, First Name:
Address:

Tel/Fax/E-mail:

Horseowner:
Name, First Name:
Address:

Tel/Fax/E-mail:

Number of samples:______ Date sampling:_________
☐ Individual sample ☐ Composite sample

Number of horses in stock:_______________________
Number of foals in stock:_________________________

Name/Mark of the horse: ________________________
Sex: ☐ m ☐ f
Age: ____
Date of the last deworming:___________ Used drug:_________________

Signs of illness: ☐ diarrhea ☐ cough
Other:__________________________________

Comments:
